# Similarity-based reasoning in conceptual spaces

**DOI:** 10.3389/fpsyg.2023.1234483

**Published:** 2023-09-05

**Authors:** Igor Douven, Steven Verheyen, Shira Elqayam, Peter Gärdenfors, Matías Osta-Vélez

**Affiliations:** ^1^IHPST / CNRS / Panthéon–Sorbonne University, Paris, France; ^2^Erasmus School of Social and Behavioural Sciences, Erasmus University Rotterdam, Rotterdam, Netherlands; ^3^School of Applied Social Sciences, De Montfort University, Leicester, United Kingdom; ^4^Cognitive Science, Lund University, Lund, Sweden; ^5^Institute of Philosophy II, Ruhr University Bochum, Bochum, Germany

**Keywords:** category-based induction, conceptual spaces, inference, similarity, similarity based reasoning

## Abstract

Whereas the validity of deductive inferences can be characterized in terms of their logical form, this is not true for all inferences that appear pre-theoretically valid. Nonetheless, philosophers have argued that at least some of those inferences—sometimes called “similarity-based inferences” —can be given a formal treatment with the help of similarity spaces, which are mathematical spaces purporting to represent human similarity judgments. In these inferences, we conclude that a given property pertains to a category of items on the grounds that the same property pertains to a similar category of items. We look at a specific proposal according to which the strength of such inferences is a function of the distance, as measured in the appropriate similarity space, between the category referenced in the premise and the category referenced in the conclusion. We report the outcomes of three studies that all support the said proposal.

## Introduction

Decades of research in thinking and reasoning have taught philosophers and psychologists alike that the richness and variety of human inference is far from being matched by the formal systems meant to capture this inference. For example, deductive logic focuses on inferences whose validity can be characterized in terms of *form*, such as

Alice is a philosophy professor
All philosophy professors are nice
Alice is nice

which is valid because it is an instance of the schema

*Pa*
∀*x*:*Px*⊃*Qx*
*Qa*

The validity of inferences of this schematic form is guaranteed by the fact that the set we designate by the predicate *P* is included in the set we designate by the predicate *Q*, so that any object to which the first predicate applies is one to which the second applies as well.

It is well-known, however, that not every inference that appears pre-theoretically valid is warranted due to its form. Famous cases include

This vase is blue....
This vase is colored

and

This vase is blue all over
This vase is not red

To account for inferences like these, it is actually possible to stick to deductive logic if we are willing to supplement it by meaning postulates (so that, e.g., being blue implies being colored in virtue of the *meanings* of “blue” and “colored”), in the manner of Carnap (1952). But even then, there remain forms of inference that appear perfectly fine but that escape analysis in terms of logical form.

This paper will be concerned with inferences that appear valid not in virtue of their form, not even in virtue of their form together with meaning postulates, but in view of certain similarity relations connecting their premise(s) and conclusion. In psychology, much of the relevant work comes under the heading of “category-based induction.” To illustrate, suppose you know a lot about cats. Among other things, you know that they are prone to developing kidney problems when they grow older. Now compare these statements:

1. Dogs are prone to developing kidney problems when they grow older.
2. Elephants are prone to developing kidney problems when they grow older.

Suppose you know little about dogs or elephants and nothing about what diseases they are prone to developing. Still, given what you know about cats, you are probably more confident in 1 than in 2, because dogs appear much more similar to cats than elephants do. That, at least, is what the current data about category-based induction suggest (e.g., Rips, 1975; Osherson et al., 1990).

Pioneering philosophical work on this type of inference is to be found in Carnap (1980), where it is discussed under the label of “reasoning by analogy on the basis of the similarity of attributes” (p. 39). Carnap distinguished between two subtypes of analogical reasoning, *similarity-based inference* and *proximity-based inference*.^1^ The following argument exemplifies the latter type:

Alice loves *Rigoletto*..
Alice loves *La bohème*

On Carnap's analysis, one would expect people to be inclined to deem arguments of this type valid to the extent that, in their opinion, the two mentioned operas are similar to each other (see also Paris and Vencovská, 2017). Douven et al. (2022) conducted an empirical study aimed at testing this idea, finding that their participants' preparedness to infer a conclusion of the form *Lac* from a premise of the form *Lab* was indeed reliably predicted by how similar, in their participants' judgment, *b* was to *c*.

The present paper focuses on Carnap's first subtype of analogical reasoning, similarity-based inference or category-based induction, which concerns similarity relations between *classes* of items, and not, as the inference about Alice, similarity between *individual* items. More abstractly put, similarity-based inferences are of the following form:

*A*s have property *P*
*B*s have property *P*

Osherson et al. refer to these inferences as “specific,” because the categories in the premise and the conclusion reside at the same hierarchical level. The validity of such inferences is clearly *not* a matter of their form: we have no difficulty instantiating *A*, *B*, and *P* in ways which make the inference rejectable. Rather, their validity seems to depend on how similar the categories involved (*A* and *B*, in the schema) are to each other.^2^

That this form of inference relies on the notion of similarity can seem a cause for concern. How can we hope to have anything resembling deductive logic that could help us determine the validity of similarity-based inferences if, as was most forcefully argued by Goodman (1972), similarity is a vague and ill-understood notion? The response here begins by pointing out that important progress has been made in the study of similarity since Goodman published his critique. We in particular want to mention the geometric type of analysis of similarity to be found in the works of Shepard (1964, 1987), Nosofsky (1986, 1987, 1989); also Nosofsky and Zaki (2002), Gärdenfors (2000, 2014), Lewis and Lawry (2016), and others. In fact, Carnap (1980) was already aware of this geometric approach to similarity, and (to the best of our knowledge) he was the first to propose that this approach is essential to understanding similarity-based inference. A geometric approach to similarity also underlies Rips' (1975) study of inferences about natural categories, which can be regarded as an important precursor of the present work. Moreover, a version of this approach to similarity also served as the theoretical framework in Douven et al.'s (2022) study mentioned above.

In this geometric framework, similarity relations are represented in one- or multidimensional metric spaces, where the dimensions correspond to fundamental qualities that items in the domain of interest may possess and distance between the representation of items in the given space correlates inversely with how similar these items are to each other, in the respect the space is intended to model. Famous examples are the CIELAB and CIELUV color-similarity spaces, which are meant to represent the similarities between color shades as perceived by humans.^3^ Both are three-dimensional Euclidean spaces, with one dimension representing luminosity (the amount of white mixed in), a second dimension representing saturation (how “full” or “deep” the color is), and the third representing hue (roughly, where a color lies on the familiar color circle). Other examples include auditory spaces, taste space, olfactory space, various shape spaces, action and event spaces, face space, and “moral” space.^4^

Similarity spaces are commonly constructed by applying some statistical dimension-reduction technique (such as multi-dimensional scaling or principal component analysis) to a large set of similarity judgments or similar data (such as confusion probabilities or correlation coefficients; see Abdi and Williams, 2010; Borg and Groenen, 2010; Hout et al., 2013b). An alternative approach is to let participants in an experiment build a similarity space directly, by asking them to spatially arrange a number of items in a way which best reflects their similarity judgments about those items (Goldstone, 1994; Hout et al., 2013a). We have more to say about this so-called spatial arrangement approach below, as it is the one we are using in one of our studies.

Gärdenfors (2000) shows how similarity spaces can be used to represent concepts. Specifically, on his proposal concepts are convex regions in similarity spaces. For instance, the concept RED is a convex region in color space, and the concept SWEET is a convex region in taste space. There are different ways to build a conceptual space on top of a similarity space. The one best explored, and favored by Gärdenfors, first locates the prototypes of the concepts we want to represent in the space and then uses the mathematical technique of Voronoi tessellations to carve up the space into separate regions (Okabe et al., 2000). For instance, to represent the basic color concepts using CIELAB space, we only need to find the locations of the prototypes of those concepts (typical red, typical blue, and so on) in the space, and then the Voronoi tessellation generated by those points gives us the concept representations we are after. Corresponding to the previous examples of similarity spaces, there exist conceptual spaces for taste concepts, olfactory concepts, shape, action, and event concepts, moral concepts, and more.^5^,^6^

As mentioned, Carnap already had the idea of using similarity spaces to formalize similarity-based arguments, specifically, defining the validity of such arguments in terms of distances as measured in the appropriate spaces. Carnap's conception of similarity spaces was rather rudimentary, lacking the precise and detailed conceptual spaces framework as it is known nowadays. Using this framework, Osta-Vélez and Gärdenfors (2020) present a more detailed proposal for formalizing similarity-based arguments. According to these authors, the strength of a similarity-based argument depends on three things: premise–conclusion similarity, premise typicality, and conclusion typicality, in the following precise manner:


$$\log 𝔼[S{\left(X\to Y\right)}_{Z}]\;=\text{ sim}(X,Y)\;+\;a\text{ sim}(X,p^{Z})\;+\;b\text{ sim}(Y,p^{Z})$$

In words, this says that the logarithm of the expectation that *Y*s have *S* if *X*s have *S*, with *X* and *Y* designating concepts both falling in a more encompassing concept designated by *Z*, is equal to the weighted sum of the distance between *X* and *Y*, the distance between *X* and the *Z* prototype (*p*^*Z*^), and the distance between *Y* and the *Z* prototype. As these authors point out, the coefficients *a* and *b*—the weights—are free parameters that are to be estimated from the data.

Osta-Vélez and Gärdenfors illustrate their proposal by means of a bird space and a mammal space. While these illustrations are helpful, the authors note that the spaces they appeal to are not based on any data and are made up for the occasion. Therefore, they cannot serve to show that Osta-Vélez and Gärdenfors' proposal is empirically adequate.^7^ Nevertheless, the illustrations suggest a clear plan for testing the proposal, to wit, empirically determine the structure of bird space or mammal space, use the thus obtained space to predict the strength of similarity-based inferences, and then check empirically the accuracy of those predictions.

In this paper, we test Osta-Vélez and Gärdenfors' proposal precisely in this way, that is, we construct a mammal space and use that to predict the inference strength of similarity-based inferences concerning mammals. The predictions are then compared with people's judgments of the strength of those inferences.

Previous research had cast doubt on the relevance of prototypical information (Douven et al., 2022). There are also theoretical doubts about the existence of a mammal prototype (Malt, 1995; Taylor, 1995; Voorspoels et al., 2011a,b). This was reason to simplify the hypothesis to: The strength of similarity-based inferences is a function of the distance between the premise category and the conclusion category as measured in the relevant similarity space. In other words, if we know how distant one type of mammal is from another type of mammal in a person's mammal space, then we are able to predict how strongly that person will agree that if the former has a given property *P*, then so has the latter.

We thus aim to test whether premise–conclusion similarity in inductive reasoning can be modeled in the conceptual spaces framework. The more similar premise and conclusion are in the conceptual space, the stronger the argument should be. We report three studies aimed at testing this hypothesis. Study I aims to arrive at an appropriate mammals space and explore its predictive value for single-premise arguments. Study II is a pre-registered replication of Study I with more participants as well as items and which also removes a potential confound. Study III explores whether the findings from Studies I and II can be extended to multi-premise arguments. Studies I and II are indebted to the early work of Rips (1975), who was the first to empirically relate distances in conceptual space to inductive strength. Study III is indebted to Osherson et al. (1990), who were the first to study premise–conclusion similarity in multi-premise arguments, though they did not understand similarity in a geometrical fashion.

The reported studies were approved by the Ethics Review Committee of the Department of Psychology, Education, and Child Studies of Erasmus University Rotterdam (application #20-060a).

## Study I

###  Method

#### Participants

Participants were 83 undergraduate psychology students from Erasmus University Rotterdam; they took part in return for credits. After removing participants who had missed at least one of three attention checks or who indicated that they had been diagnosed with dyslexia, as well as removing one participant who indicated that they could not properly move items in one of the two tasks (see below), there were 58 participants left for the analysis.^8^ Their mean age was 20.88 (± 2.21); 16 were male, 42 female. They all indicated their English reading ability to be at CEFR level B2 or above on the Council of Europe's self-assessment grid.

#### Materials and procedure

The study was run online using the Qualtrics platform (https://www.qualtrics.com/). It consisted of two parts, the first presenting a Spatial Arrangement Task to help build, per participant, a mammal space, which was hoped to reflect the participant's similarity perceptions regarding twenty mammals. The twenty mammals were randomly selected from Henley's (1969) work on similarity among mammals. One approach would have been to elicit pairwise similarity judgments and then build a space from those using a multi-dimensional scaling technique. However, for twenty mammals, each participant would have had to make $\left(\frac{20}{2}\right)=190$ similarity judgments, which would have made the study overlong. That is why we opted for the Spatial Arrangement Task, which has recently become available as a functionality on Qualtrics (Koch et al., 2020). Because of its spatial nature, the arrangement task is a natural task to obtain geometric similarity representations (Verheyen et al., 2016, 2022). Spatially arranging 20 items in terms of similarity has also been found to be about 2.5 times faster than providing 190 pairwise similarity judgments and participants accordingly judge the arrangement task less tiresome, but nevertheless still challenging since in positioning an item, one needs to take its distance to all other items into account (Verheyen et al., 2022).

Specifically, the first task presented participants with a screen containing the names of twenty mammals, randomly grouped in two columns of ten in the middle of the screen. Participants were told that they could drag any of the names to any location on the screen they wanted, and they were instructed to rearrange all of them in such a way that the resulting constellation would reflect the animals' similarity. More exactly, participants were asked to use the whole screen and to make sure that more similar animals were placed closer together and more dissimilar animals further apart. Participants had to move all animal names from their initial position and confirm that they were satisfied with the resulting configuration before they could continue to the second task.

In the second task, participants were asked to indicate the strength of thirty similarity-based inferences. For each participant, thirty pairs were randomly drawn from the $\left(\frac{20}{2}\right)\times 2$ (because order matters) = 380 possible pairs of mammal names that can be selected from the stimuli used in the first task, and for each pair, the participant was asked to suppose that mammals denoted by the first member of the pair had a certain property (which was only specified abstractly as a random combination of a letter and a digit, such as K7 or I3) and was then asked how strongly it followed from that supposition that mammals denoted by the second member of the pair of names had the same property. The response had to be given by positioning a slider on a scale going from 0 to 100%, with the former anchor being additionally labeled “Does not follow at all” and the latter being labeled “Follows very strongly.” For instance, the participant could be asked to suppose that cats have property M4 and then be asked to indicate, in the way just described, how strongly in their opinion it followed that zebras have property M4. This task started off with a practice question, which read as follows:

Suppose elephants have property Q2. Then how strongly does it follow that bears have property Q2? Please rate the statement by moving the slider to the left or the right. By “follow” we mean that the context as described invites this conclusion. For example, if you think that the assumption that elephants have property Q2 definitely invites the conclusion that bears have that property too, then move the slider to the right. You are encouraged to consider the full range of the scale, including low, intermediate, and high levels, such as 37 %, 58 %, and 82 %, respectively.

After participants had answered the practice item, they proceeded to the thirty actual study arguments. Each argument was presented on a new screen with the slider positioned on the middle value (50 %) by default.

###  Results and discussion

**Figure 1:**
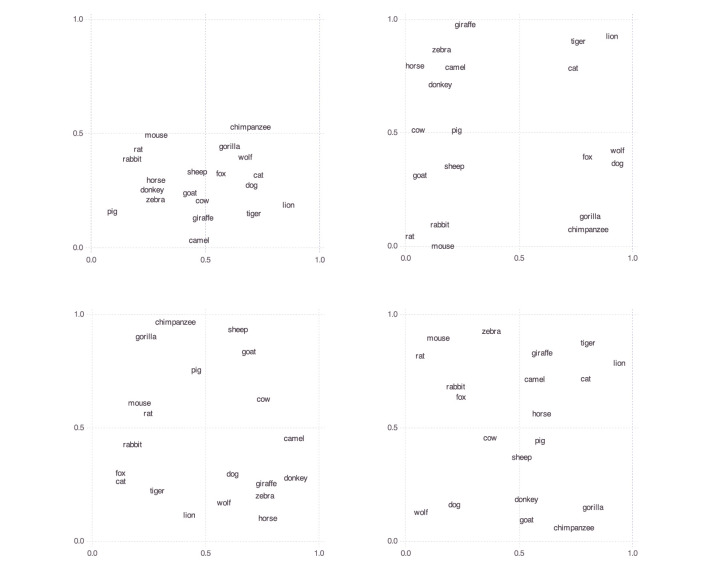
Personal mammal spaces of four randomly chosen participants.

For each participant, the arrangement task provided us with the coordinates of each label on the participant's screen and also with the size of the screen, so that all results could be scaled to the unit square. We interpreted the scaled results as the participants' personal mammal spaces and the distances between pairs of mammal names in those spaces as indicating the participants' pairwise similarity judgments.^9^ Figure 1 shows these spaces for four randomly selected participants.

Having at hand a personal mammal space for each participant, we could ask to what extent the distances in a participant's space predict, for each similarity-based argument the participant had evaluated in the second task, how strongly, according to that participant, the conclusion followed from the premise. To answer this question, we ran a regression analysis per participant, with inference strength judgments as response variable and Euclidean distances as measured in the participant's mammal space as predictor variable. More exactly, in each model we ran, the dependent variable consisted of thirty data points, constituted by the participant's judgments of inference strength of whichever thirty arguments they had been presented with in the second task, and the independent variable consisted of thirty data points as well, each being the distance in their personal mammal space between the mammals referred to in the corresponding argument's premise and conclusion.

**Figure 2:**
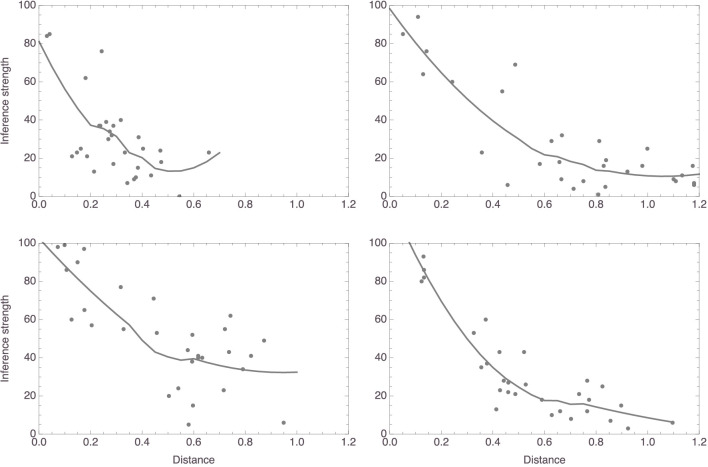
Data plots for the four participants whose mammal spaces are shown in Figure 1 (with corresponding panels showing data for the same participant), with smoothers added to highlight trends.

Figure 2 plots, for four random participants, distances against judgments of inference strength. In all four, there is a clear relation between the two variables, as highlighted by the added smoothers. These plots already suggest that the data are probably better analyzed using nonlinear regressions than using linear regressions, a suggestion that is reinforced by inspecting the corresponding plots for the other participants. This was in fact to be expected in light of work by Shepard (1964, 1987), Nosofsky (1986, 1987, 1989), and others, which suggested that similarity is a monotonically decreasing, but not strictly a linear, function of distance in a similarity space. Specifically, these authors successfully modeled similarity as an exponentially decaying function of distance. Accordingly, we fitted models of the form *f*(*x*) = *c*_1_×exp(−*c*_2_*x*), always with measured inference strength as the response variable and distance in mammal space as the predictor. For this study as well as for the studies to be reported below, readers are encouraged to consult the supplementary *Mathematica* notebook, where all results are presented in full detail. As for this study, it can be seen in the notebook that the nonlinear models gave more satisfactory results than linear ones, which we also fitted. Here, we only report the outcomes from the nonlinear model fits.

**Figure 3:**
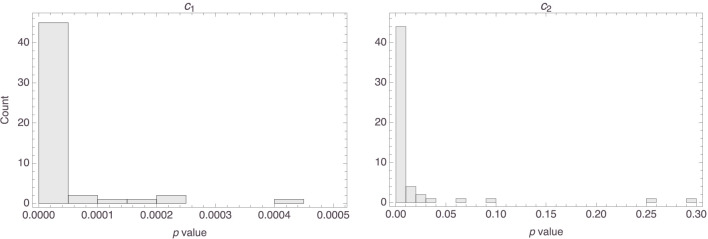
Histograms of the *p* values of the parameters in the 58 individual models.

Figure 3 shows histograms of the *p* values that were obtained for the two parameters, *c*_1_ and *c*_2_. It is clear that, for most participants, both parameters were highly statistically significant. Indeed, the median *p* value of the first parameter in the 58 models was basically 0 (MAD = 0), and the median *p* value for the second parameter was 0.0002 (MAD = 0.0002).

**Figure 4:**
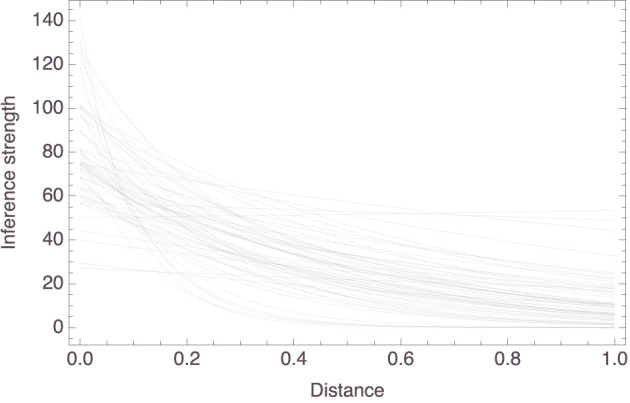
Graphical presentation of the 58 individual models.

**Figure 5:**
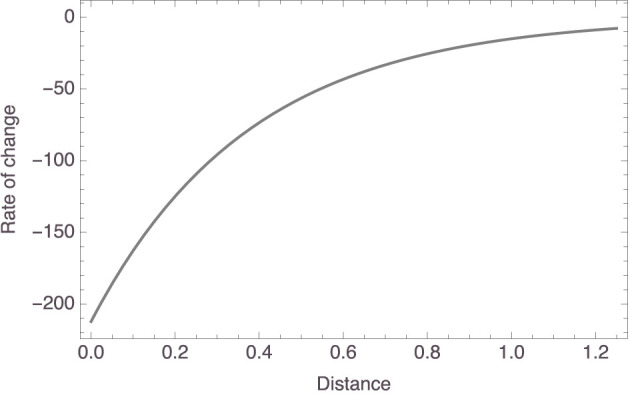
Plot of the derivative of the “average” model.

Figure 4 plots the individual models. The trend is clear: a participant's strength rating for an inference from “*X*s have *P*” to “*Y*s have *P*” *decreases* as the distance between *X* and *Y* in that participant's mammal space *increases*. To be more exact, the *c*_1_ parameter had a mean value of 80.01 (±23.99) and the *c*_2_ parameter had a mean value of 2.65 (±2.10). So, for the “average” participant, the relationship between inference strength and premise–conclusion distance is given by *f*(*x*) = 80.01 × exp(−2.65*x*). To facilitate interpretation, note that this function has the derivative *f*′(*x*) = −212.23 × exp(−2.65*x*), whose graph on the relevant domain is shown in Figure 5. It is seen that, for small premise–conclusion distances, a tiny increase in that distance already leads to a sharp drop off in inference strength, while the effect of tiny differences in distance diminishes as the premise–conclusion distance increases.

**Figure 6:**
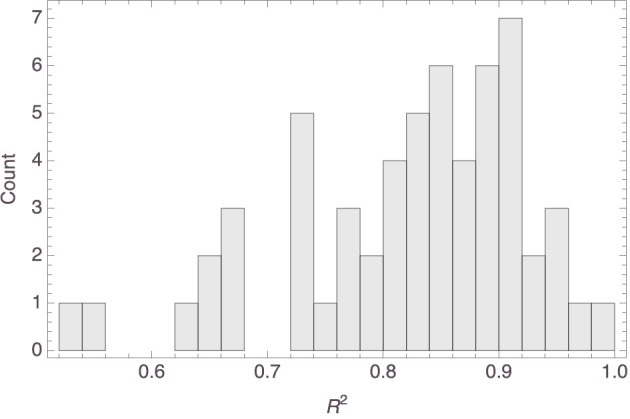
Histogram of the *R*^2^ values of the models visualized in Figure 4.

Figure 6 shows a histogram of the *R*^2^ values of the individual models. It is seen that the model fit was mostly satisfactory to excellent. In fact, the median *R*^2^ value was 0.85 (MAD = 0.06). While the fit could have been better still, it is to be noted that the Spatial Arrangement Task with twenty items to be arranged is hard. This was not just our own experience pre-testing the survey; the claim is supported by the observations in Verheyen et al. (2022), where participants indicated the task to be more challenging the more items needed to be arranged. Indeed, early work on the Spatial Arrangement Task already raised the concern that some participants interpret it as a sorting task or only arrange the items with respect to a subset of reference stimuli, thereby not taking all pairwise relations into account (Goldstone, 1994; Hout et al., 2013a; Verheyen et al., 2016). It is also known, however, that aggregating individual arrangements does away with many of these idiosyncracies and tends to yield similarity spaces that are in line with spaces obtained through pairwise similarity judgments (Richie et al., 2020; Verheyen and Storms, 2021; Verheyen et al., 2022). This made us decide to construct an aggregate mammal space from the individual mammal spaces, hoping that the construction would establish a kind of regression to the mean and thereby filter out some noise probably attributable to working memory limitations.

**Figure 7:**
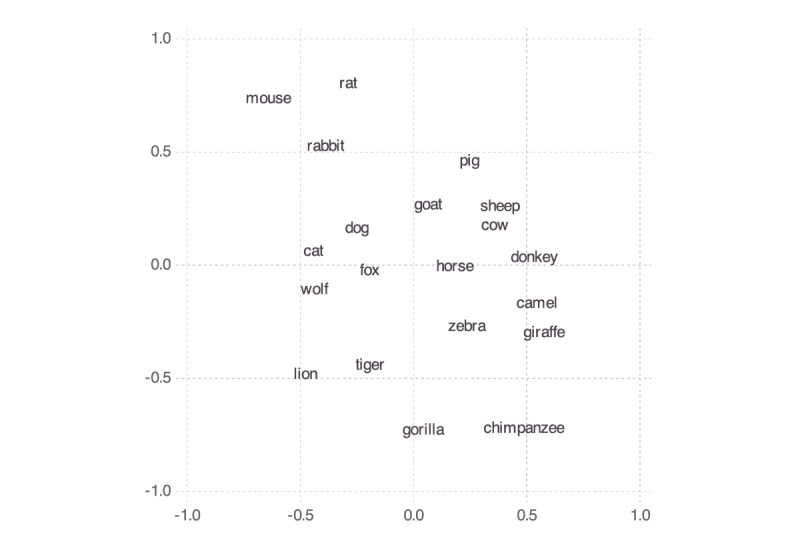
Mammal space aggregating personal mammal spaces.

**Figure 8:**
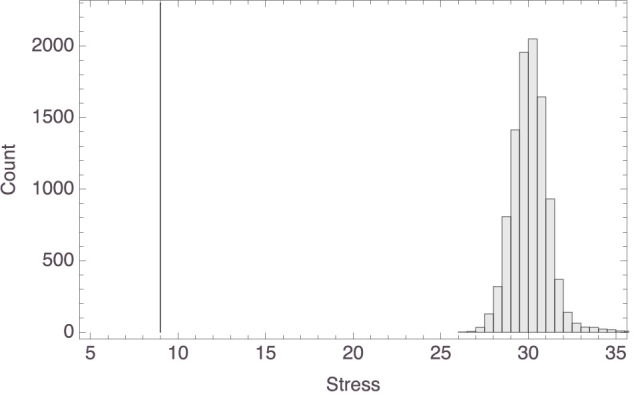
Histogram of the stress values of multi-dimensional scaling models based on random distance matrices, with the stress value of the actual model (8.99) marked by the gray vertical line.

To arrive at this aggregate space, we started by (again) supposing that the participants' Spatial Arrangement Task responses indicated their similarity judgments. Because in those responses only relative distances among labels (containing names of species) mattered, and so for instance orientation of the space did not matter, we cannot simply obtain an aggregate space by averaging across participants' *x* and *y* coordinates for any given animal. Instead, we averaged the Euclidean distances among the items and then applied classical multi-dimensional scaling to those, assuming the Euclidean distance function and the strain loss function, which yielded the space shown in Figure 7.^10^ The resulting model had a stress of 8.99, which counts as good. We also compared this stress value with the stress values of 10,000 multi-dimensional scaling models (with the output dimensions set to 2) obtained from random distance matrices. The lowest (i.e., best) stress value found among those models was >26. The data from the comparison are shown in Figure 8. This supports the conclusion that we can safely interpret the mammal space in Figure 7 and the corresponding inter-exemplar distances and in particular that the latter do not represent random data.

We were interested in the effect of replacing in the previous nonlinear models the predictor variable, which for any given participant consisted of distances measured in that participant's personal space, with distances as measured in the aggregate space. We found that model fit was indeed somewhat better for the distances based on the aggregate space, as the median *R*^2^ value was now 0.87 (MAD = 0.05). Here, too, the parameters were statistically significant for virtually all participants, the median *p* values (as well as the corresponding MADs) being essentially 0.

**Figure 9:**
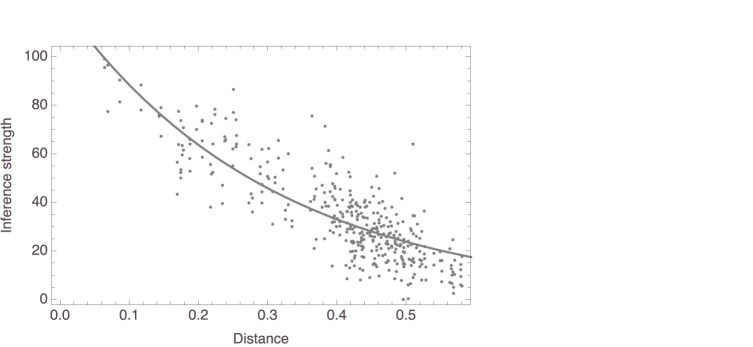
Exponential model of mean responses to inference strength task regressed on distances in aggregate space (shown with data).

Finally, we also aggregated the data obtained in the inference strength task, averaging, for each question, the responses given by the participants that had been presented with that question. This yielded 378 data points, given that the questions for two of the 380 possible pairs of mammal names had not been presented to any of the participants who were left for the analysis. On average, each pair received 4.58 (± 2.17) responses. The model we fit to the average responses again had the form *f*(*x*) = *c*_1_×exp(−*c*_2_*x*). This model, too, revealed distance to be a significant predictor of inference strength, with *c*_1_ = 122.70, SE = 4.14, *t* = 29.62, *p* < 0.0001, and *c*_2_ = 3.28, SE = 0.10, *t* = 31.83, *p* < 0.0001. Figure 9 shows the model, together with the data. For this model, *R*^2^ = 0.93.

In sum, our hypothesis was that the strength of an inference from “*X*s have property *P*” to “*Y*s have property *P*” can be predicted on the basis of how similar *X* and *Y* are, where this similarity is formalized as the distance between these concepts in the appropriate space. This hypothesis is clearly supported by the data, in that participants' judgments of inference strength could be reliably predicted from their personal mammal space. Creating an aggregate space from the personal space made sense in view of the difficulty of the Spatial Arrangement Task, and we indeed got even better predictions on the basis of that aggregate space.

## Study II

The second part of the first study had been limited to thirty questions. We conducted a further study rerunning the inference strength part of the first study but now expanding the number of questions from thirty to fifty. That the aggregate space constructed in the analysis of the first study had given somewhat better results in the regressions as compared to the individual spaces warranted analyzing the results from the new study using the same aggregate space, meaning that there was no need to rerun the first part of the first study. This also eliminated any risk of carry-over effects that was attached to the first study. This study was preregistered, following the procedure and analysis plan of Study I.^11^

###  Method

#### Participants

Participants were 132 undergraduate students in psychology from Erasmus University Rotterdam, who had not taken part in Study I. We used the same exclusion criteria as in the first study, which left us with 110 participants for the analysis. The average age of these participants was 20.25 (± 1.83); 10 participants were male, 99 female, and one participant preferred not to say. The English reading ability of all participants was at CEFR level B2 or above.

#### Materials and procedure

The materials and procedure were the same as for the second part of Study I, with the exception that now each participant was asked fifty questions instead of thirty.

### Results and discussion

We fitted again, for each participant individually, an exponential model of the same form that was used throughout the analysis of the first study, with the participant's responses to the inference strength questions as response variable and the distances among the corresponding pairs referenced in the questions as measured in the aggregate space from Study I as predictor variable.

**Figure 10:**
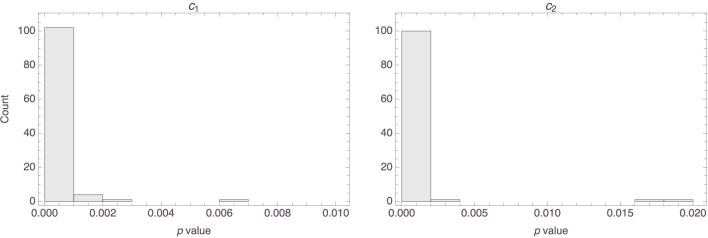
Histograms of the *p* values of the parameters in the 110 individual models.

**Figure 11:**
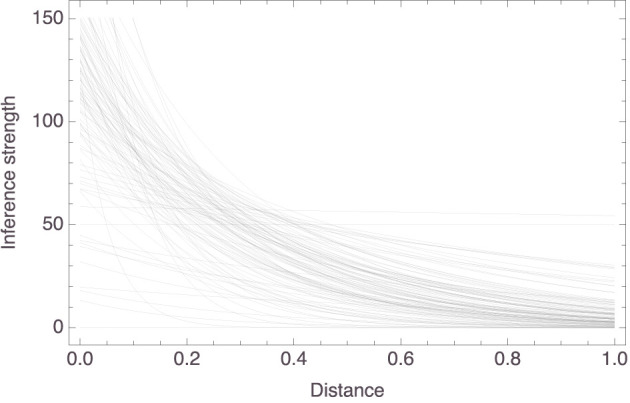
Graphical presentation of the 110 linear models with the predictor based on the aggregate space from Study I.

**Figure 12:**
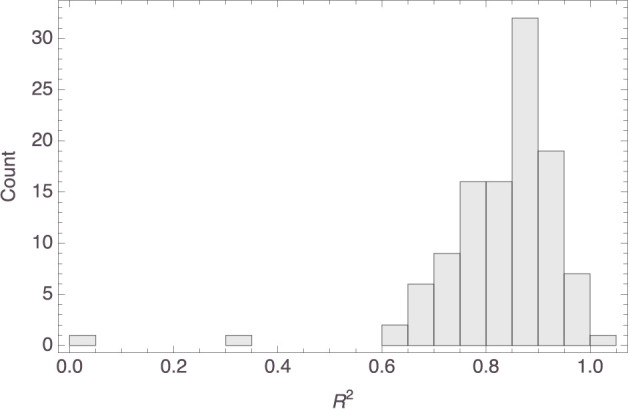
Histogram of the *R*^2^ values of the models visualized in Figure 4.

Here, too, it was found that the parameters reached statistical significance in all models, the median values (as well as the corresponding MADs) being essentially 0 for both parameters; see Figure 10 for histograms of the *p* values. As Figure 11 shows, in most models inference strength decreased rapidly with increasing distance, again consistent with what we found in the previous study. Figure 12 shows a histogram of the *R*^2^ values for these models; their median value was 0.86 (MAD = 0.06). Further details about the models are to be found in the Supplementary material.

**Figure 13:**
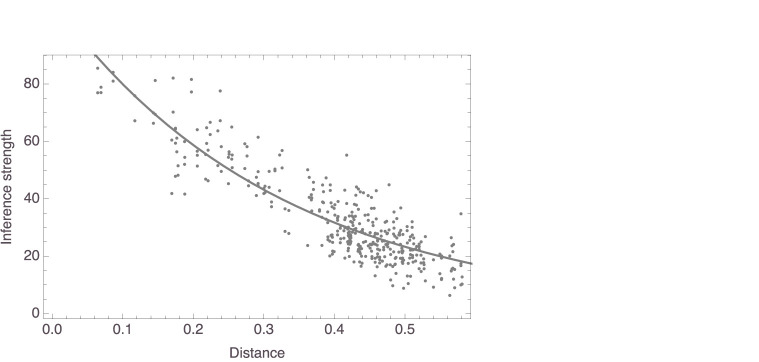
Exponential model of aggregate responses to the inference strength task regressed on aggregate distances (shown with data).

In this analysis, we also aggregated the inference strength responses, in the same way we did this in the first study. This now gave 380 data points, given that all questions had received responses from at least some of the participants left for the analysis, the average number of responses for a pair being 14.47 (± 3.51). The average responses were again regressed on the distances between the corresponding pairs of mammals, as measured in aggregate space. We found here as well that distance reliably predicted average inference strength, with *c*_1_ = 108.75, SE = 2.74, *t* = 39.69, *p* < 0.0001, and *c*_2_ = 3.08, SE = 0.08, *t* = 40.96, *p* < 0.0001. See Figure 13 for a visualization of the model, which had an *R*^2^ value of 0.96.

Thus, the results from the second study provided further evidence for our hypothesis that the strength of similarity-based arguments can be reliably predicted from distances in the space which represents the kinds referenced by the argument's premise and conclusion.

## Study III

Studies I and II only looked at single premise arguments, which is also what our main hypothesis pertains to. But, of course, the role of similarity in reasoning is not limited to such arguments. For instance, in assessing an argument like this,

Cows have sesamoid bones
Horses have sesamoid bones
Sheep have sesamoid bones

we arguably take into consideration how similar the premise categories are to the conclusion category. Arguments of this kind have been studied by Osherson et al. (1990), who were able to reliably predict inference strength on the basis of (i) how similar to the conclusion category the most similar premise category is, and (ii) the “coverage” of the premise categories, which is a kind of average perceived similarity between the premise categories and other relevant categories (see below for a precise definition). While Oshershon et al. measured similarities by having their participants rank order pairs of items, we are specifically interested in whether it is possible to account for the perceived inference strength of multi-premise category-based inductions within the conceptual spaces framework. In addition, whereas Osherson et al. only investigated arguments pertaining to one conclusion category (i.e., horse), we study inferences across a wide range of premise–conclusion combinations. Osta-Vélez and Gärdenfors (2020), which inspired the previous studies, do discuss multi-premise category-based induction, but their theoretical proposal—involving convex hulls—is not readily applicable to our materials. So, we consider this study to be more exploratory and want to be open to various hypotheses concerning the role similarity plays in the said type of arguments. Next to Oshershon et al.'s hypothesis, for which they reported support, we also want to consider the possibility that, in our framework, both premises play an equal role in determining inference strength, as well as the possibility that both play a role together with their coverage, in the sense of Osherson et al., or perhaps together with how far apart they are in the space (i.e., a different sense of coverage than that in Osherson et al.).

###  Method

#### Participants

Participants were 166 undergraduate students in psychology from Erasmus University Rotterdam, who had not taken part in either of the previous studies. We used the same exclusion criteria as previously, which now left us with 139 participants. The average age of these participants was 21.50 (± 3.42); 29 participants were male, 108 female, and two participant preferred not to say. The English reading ability of all participants was at CEFR level B2 or above.

#### Materials and procedure

As in the second task of the first study, and as in the second study, participants were asked to indicate the strength of similarity-based inferences. The main difference now was that each argument had *two* premises. Because this made the task more complex, participants were presented with forty instead of fifty arguments, as were the participants from Study II. Specifically, we drew, randomly for each participant, forty triples from the 3,420 possible triples of mammal names that can be selected from the stimuli used in the previous studies, and for each triple, we asked the participant to suppose that mammals denoted by the first member of the triple had a given property (again specified abstractly) as well as that mammals denoted by the second member of the triple had that same property, and then asked the participant how strongly it followed from those suppositions that mammals denoted by the third member of the triple of names had the given property. Responses had to be given in the same way as before.

###  Results and discussion

Because this study had an exploratory character, we looked at a range of models, all having inference strength as the response variable. While, as said, Osta-Vélez and Gärdenfors (2020) are silent about multi-premise category-based inductions, the most obvious extension of their proposal suggested to consider the distance between, on the one hand, the premise-categories and the conclusion-category—to be designated as δ(P1, C) and δ(P2, C)—as main candidates for predicting inference strength. A priori, it also seemed to make sense to look at the “third” distance, that is, the distance between the premise-categories, δ(P1, P2), as a possible predictor. Functions of the said measures (e.g., the mean of the premise–conclusion distances) were prima facie candidates as well.

A different set of possible predictors was suggested by Osherson et al.'s (1990) paper. As mentioned previously, these authors studied multi-premise category-based inductions and found that (i) the maximum of the similarities between, on the one hand, the premise-categories and, on the other, the conclusion-category, and (ii) the coverage of the premise-categories to be reliable predictors of inference strength. In the conceptual spaces framework, which we are assuming, the former amounts to the minimum of δ(P1, C) and δ(P2, C), for any given triple of premise-categories and conclusion-category. And for a given mammal space, with δ the Euclidean distance defined on that space, the coverage of a pair of premises P1 and P2 is the average of the set of values


{​min(δ(P1,C),δ(P2,C)):C∈𝒞}

with *C* the class of mammal concepts from our materials. Less formally, for each concept C, take the minimum of δ(P1,C) and δ(P2,C), and then average all those minima; that average is the coverage of P1 and P2.

Starting with the predictors suggested by Osta-Vélez and Gärdenfors' work, fitting linear models with δ(P1, C), δ(P2, C), and δ(P1, P2) as predictors led to disappointing results. Moving again to nonlinear regression analysis, we obtained the best results for a model of the form *f*(*x, y*) = *c*_1_×exp(−*c*_2_*x*)+*c*_1_×exp(−*c*_2_*y*), where the only predictors were δ(P1, C) and δ(P2, C). In all models which included these predictors, or even just one of them, adding δ(P1, P2) as a further predictor, whether as a linear, a quadratic, or an exponential term, only rarely improved model fit. Moreover, the added predictor typically failed to reach statistical significance. Also, using separate pairs of coefficients for the two predictors not only led to convergence failure for a number of participants but also did not lead to any improvements for those participants for whom convergence *was* reached.

For the predictors based on Osherson et al.'s work, we also found nonlinear models to outperform linear ones. The best nonlinear models we were able to find had the form $f\left(x,y\right)=c_{1}\times \exp\left(-c_{2}x\right)+y^{2}$, with the minimum of δ(P1, C) and δ(P2, C) and, respectively, coverage as predictors.

**Figure 14:**
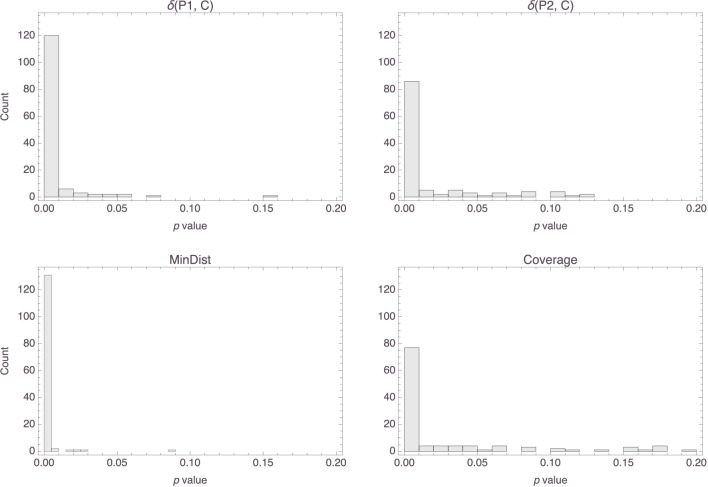
Histograms of the *p* values for the parameters in the Osta-Vélez and Gärdenfors based models **(top row)** and in the Osherson et al. based models **(bottom row)**.

**Figure 15:**
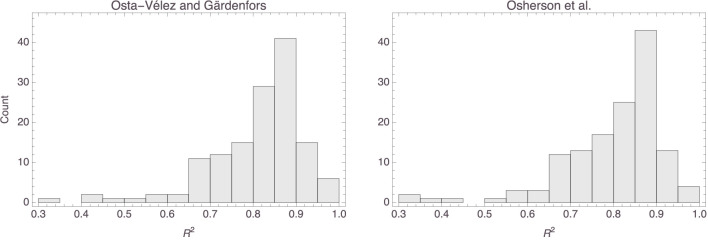
Histograms of the *R*^2^ values for the Osta-Vélez and Gärdenfors based models **(left)** and the Osherson et al. based models **(right)**.

The top row of Figure 14 shows histograms of the *p* values that were obtained for the parameters in the Osta-Vélez and Gärdenfors based models, and the bottom row does the same for the Osherson et al. based models. The median *p* values for both parameters in the former models were around 0.0002 (the associated MADs were both around 0.0003), while those for the parameters in the latter models were essentially 0 (MAD = 0) for the first and 0.003 (MAD = 0.003) for the second. Model performance also tended to be more than satisfactory for both types of models, the median *R*^2^ value for both being 0.84 (MAD = 0.05 for the Osta-Vélez and Gärdenfors models and MAD = 0.06 for the Osherson et al. models). Figure 15 shows a histogram of the *R*^2^ values obtained for the two types of models.

**Figure 16:**
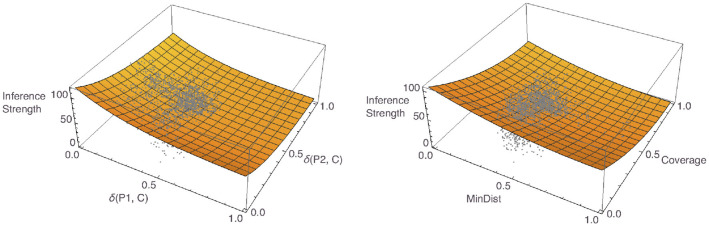
Plots of the best Osta-Vélez and Gärdenfors based aggregate model **(left)** and the best Osherson et al. based aggregate model **(right)**, with data overlayed.

Finally, we aggregated again the responses to the inference strength questions, which in this case yielded 2,763 data points. In this study, too, not all questions had received responses, the average number of responses for a pair being 1.63 (± 1.26). We fitted a number of different models to these responses. The best model had δ(P1, C) and δ(P2, C) as predictors and had the same form as was used in the per-participant analyses: *f*(*x, y*) = *c*_1_×exp(−*c*_2_*x*)+*c*_1_×exp(−*c*_2_*y*). The left panel of Figure 16 shows a plot of the model, together with the data. For the Osherson et al. based predictors, the best model we were able to find had a different form than the one we established for the individual data: $f\left(x,y\right)=c_{1}\times {\left(1-x\right)}^{2}+c_{2}\times {\left(1-y\right)}^{2}$. For a plot of the model, together with the data, see the right panel of Figure 16. The former model was superior across all model comparison criteria, with an *R*^2^ value of 0.81 vs. 0.79 for the Osherson et al. model, with an AIC value of 24,749.6 vs. 24,901.7, and with a BIC value of 24,767.4 vs. 24,919.4.

To sum up, we found that for multi-premise category-based inductions distances in mammal space could also be reliably used to predict inference strength judgments. While this study had an exploratory character, there was at least a type of model that appeared a good candidate for testing in view of Osta-Vélez and Gärdenfors' work as well as in view of the results from the first two studies. It showed that specifically the distances between, on the one hand, the premise-categories and, on the other, the conclusion-category reliably predicted inference strength. The distance between the premise-categories appeared to have little to no predictive value. Finding further inspiration in Osherson et al. (1990), we also looked at models that had as a predictors the equivalents in our mammal spaces of the predictors these authors had successfully used to predict the strength of multi-premise category-based inductions. In our analysis, these came our as significant predictors as well, though the overall results for the models using these predictors were less satisfactory than those for the former class of models.

## General discussion

This paper focused on a type of argument that projects a property from one or more classes of items onto another class of items, based on the similarity between the classes designated in the premise or premises and the class designated in the conclusion. Whereas it was known from the literature that the strength of such arguments was a matter of *how* similar the classes of items referenced in the argument are, most of that literature had treated similarity as an intuitive, informal notion, with the exception of Rips (1975). Taking our cue from ideas advanced by Carnap (1980) and Osta-Vélez and Gärdenfors (2020), we hypothesized that argument strength could actually be predicted from measured distances in a mathematical space representing similarity relations.

To test this hypothesis, we conducted three studies. In the first, participants had to complete two tasks, one asking them to construct their personal mammal space, the other asking them to judge the strength of thirty similarity-based arguments. The data we obtained from this study allowed us to fit a model per participant, with distances in the participant's mammal space as predictor and their inference strength judgments as response variable. For the vast majority of participants, distances in mammal space reliably predicted inference strength judgments, thereby confirming our hypothesis. The results were even better when instead of the personal spaces we used distances in an aggregate space.

The second study was a replication of the inference strength task from Study I, but with a larger sample of participants who now had to judge the strength of fifty arguments, a larger pool of items, without them being potentially influenced by a preceding similarity task. We fitted again a model for each participant, with the predictor variable coming from the aggregate space constructed as part of the analysis of the first study. Distances as measured in that space again proved to reliably predict inference strength judgments, yielding further evidence for our hypothesis.

Whereas the first two studies had focused on single-premise arguments, the third study looked at two-premise arguments. The findings were in line with those obtained for the single premise arguments, in that distances in mammal space could again be reliably used to predict participants' inference strength judgments. The findings from Studies I and II confirm earlier ones established by Rips (1975), who was the first to show that conceptual spaces can be used to model premise conclusion similarity in inductive arguments. The findings from Study III extend those of Osherson et al. (1990) in that premise–conclusion similarity in multi-premise arguments is also shown to hold when similarity is captured in a geometrical fashion. The results from the three studies also extend the work of Rips (1975) and Osherson et al. (1990) in that these relationships are shown across the entire range of category exemplars and similarity relations, rather than a selected subset.

More generally, the results reported in Douven et al. (2022) were already evidence that the conceptual spaces framework can serve to explain in a formal manner patterns of non-deductive reasoning that many believed to be beyond formalization. That paper looked at a type of similarity-based arguments which infer the possession of a given property by an individual from the possession of a similar property by that individual. The new data are evidence that the same framework is useful also in explaining a different type of similarity-based inferences, to wit, inferences from one or more classes of items having a given property to a similar class of items having that same property. The latter type of inferences were the subject of Osta-Vélez and Gärdenfors (2020), which formed the direct inspiration for the present work.

A point made in Douven et al. (2022) that we would like to reiterate here concerns the normative status of similarity-based inferences. Philosophers working on non-deductive logics are generally motivated by the thought that it must be possible to have norms for non-deductive forms of reasoning similar to the ones we have for deductive reasoning. There is widespread agreement, however, that so far no one has been able to pin down the former (e.g., Carnap, 1980; Maher, 2001; Bartha, 2010; Douven, 2022). Nevertheless, our theoretical work suggests that, at least for similarity-based inferences, norms of correctness can be derived from recent work on conceptual spaces, arguing that their structure is subject to rationality criteria. In particular, Douven and Gärdenfors (2020) argue that the concepts that have a place in our talking and thinking are the ones represented by optimally designed spaces. Following a suggestion already made in Douven et al. (2022), a similarity-based inference of the kind considered in this paper can be said to be warranted to the extent that the class of items designated in the premise falls under a concept that lies close to the concept under which the class of items designated in the conclusion falls, provided the space in which the concepts are represented is optimally partitioned.

Finally, we mention a limitation of the studies reported in this paper. As noted, using the Spatial Arrangement Task to obtain personal mammal spaces had a clear advantage over eliciting pairwise similarity judgments and using those to fit a participant's mammal space via multi-dimensional scaling: given the number of pairwise similarity judgments that would have been required, the latter method would have taken very long and might have yielded noisy judgments because of participants becoming inattentive, tired, or bored (Hout et al., 2013a; Koch et al., 2020). On the other hand, the Spatial Arrangement Task also has a clear disadvantage: it forces a participant's similarity space (in our case, the participant's mammal space) to be two-dimensional, where a multi-dimensional scaling of pairwise similarity judgments could have indicated that the best fit is obtained for a three- or even four-dimensional space (Verheyen et al., 2016, 2022). Given that it is now relatively easy to let web users manipulate objects in three-dimensional spaces, it should only be a matter of time before a three-dimensional Spatial Arrangement Task becomes available for researchers. Once it is, it would be worthwhile rerunning our studies using that new version of the task.

An obvious avenue for future work is the study of multi-premise arguments with more than two premises.^12^ It is reasonable to expect that if the number of premises increases to the extent that it is no longer feasible to consider all premise–conclusion similarities, the relative importance of the most similar premise and/or coverage might increase. The presentation of both single-premise and multi-premise arguments to the same participants, would constitute another test of conceptual spaces' ability to capture similarity-based reasoning phenomena. Under these circumstances, one would expect stronger inferences based on multi-premise arguments than on single-premise arguments due to better coverage of the entire space. It also remains to be seen to what extent conceptual spaces can be used to model other types of non-deductive reasoning, such as general induction arguments (where the conclusion category comprises the premise categories as in an inference from mice and elephants to all mammals) and mixed induction arguments (where the conclusion category comprises some but not all premise categories as in an inference from mice and ducks to all mammals).^13^

## Author's note

The supplementary materials for this paper consists of a Julia (Bezanson et al., 2017) script and a Mathematica notebook and is available at this repository: https://osf.io/ybvuk/. (For readers who do not have access to Mathematica, we note that Mathematica notebooks can be used interactively in the free Wolfram Player, which can be downloaded from this address: https://www.wolfram.com/player/.)

## Data availability statement

The datasets presented in this study can be found in online repositories. The names of the repository/repositories and accession number(s) can be found in the article/Supplementary material.

## Ethics statement

The studies involving humans were approved by the Ethics Review Committee of the Department of Psychology, Education, and Child Studies of Erasmus University Rotterdam. The studies were conducted in accordance with the local legislation and institutional requirements. The participants provided their written informed consent to participate in this study.

## Author contributions

ID and SV: design, analysis, and writing. SE, PG, and MO-V: writing. All authors contributed to the article and approved the submitted version.

## Footnotes

^1^Because both inference types exploit similarity relations, the terminology is somewhat unfortunate. However, it should not lead to any confusion here, given that we will only be concerned with inferences of the first type.

^2^The premise and conclusion of a similarity-based inference are typically generics (see, e.g., Gärdenfors and Osta-Vélez, 2022) and so it would be wrong to state them using a universal quantifier.

^3^Which of these spaces we are to apply depends on the viewing conditions. CIELAB space is a more accurate representation of similarity judgments when these concern colored cloths or colored pieces of paper, while CIELUV space is preferable when the comparisons concern colors shown on screen. For details, see Fairchild (2013).

^4^See, for instance, Petitot (1989) on auditory spaces, Gärdenfors (2000) and Douven (2016) on shape spaces, Gärdenfors and Warglien (2012) on action spaces, Castro et al. (2013) on olfactory space, Valentine et al. (2016) on face space, and Peterson (2017) and Verheyen and Peterson (2021) on moral spaces.

^5^See the works referenced in note 4, which not only present similarity spaces but full-fledged conceptual spaces.

^6^Geometrical models of similarity have been often criticized because of alleged limitations in accounting for context effects (Tversky, 1977; see Decock and Douven, 2011, for a review). However, Gärdenfors' (2000) conceptual spaces model does not suffer from these shortcomings (Johannesson, 2002). The model accounts for the context-sensitive character of psychological similarity in terms of a weighted distance measure. Specifically, the distance measure includes weights *w*_*i*_ that modify the salience of dimension *i* in the conceptual space. When a larger value is given to a weight *w*_*i*_, the conceptual space is “stretched” along that dimension, which means that dimension *i* will become more important when determining the similarity between categories (Gärdenfors, 2000, p. 20).

^7^As these authors point out, though, their proposal receives indirect support from the fact that it is able to explain several qualitative principles that have been empirically established in the literature on category-based induction.

^8^The first attention check presented the participants with a list of hobbies and instructed them to write “I read the instructions” in the “Other” box; this check was adapted from Pennycook et al. (2014). A second attention check consisted of a number of questions about how thoroughly the participant tended to read surveys, where one of the questions gave the explicit instruction to tick the “Strongly disagree” option. And the third check asked participants at the end of the survey whether they had answered seriously, a procedure adapted from Aust et al. (2013).

^9^Assuming the latter, we could determine the extent to which the participants agreed in their similarity judgments, by calculating Cronbach's α. This turned out to be 0.96 [95 % CI (0.95, 0.97)], which is taken to indicate a very high degree of agreement. See the Supplementary material for further details. This high level of agreement among participants also justifies subjecting the average data to MDS to construct a shared space (see below).

^10^For this, we used the MASS package for the statistical programming language R (R Core Team, 2022).

^11^See https://osf.io/eu2dj. We deviated from the preregistration in that we conducted nonlinear analyses in addition to linear ones. The conclusions do not depend on this, as can be seen in the Supplemental material, in which the results of the linear analyses are documented.

^12^Another possible avenue for future research, suggested by an anonymous referee, is to look into possible practical applications of our findings, in particular also into applications of our model in recommender systems.

^13^We are greatly indebted to three anonymous referees for valuable comments on a previous version. We are also grateful to audiences at Ruhr University Bochum and at the University of Talca for stimulating questions and discussion.
